# The effect of weather on unscheduled healthcare utilisation for mental health conditions in England, 2014–2022

**DOI:** 10.3389/fpsyt.2026.1835204

**Published:** 2026-06-30

**Authors:** Richard Elson, Julii Brainard, Natalia R. Jones, Alex J. Elliot, Iain R. Lake

**Affiliations:** 1 School of Environmental Sciences, University of East Anglia, Norwich, United Kingdom; 2 National Institute for Health and Care Research Health Protection Research Unit in Gastrointestinal Infections, University of East Anglia and Newcastle University, Norwich, United Kingdom; 3 Norwich Medical School, University of East Anglia, Norwich, United Kingdom; 4 National Institute for Health and Care Research Health Protection Research Focal Unit in Outbreak Response Behaviours at Kings College in collaboration with the University of East Anglia and the UK Health Security Agency, London, United Kingdom; 5 Real-time Syndromic Surveillance Team, Field Services, Chief Medical Advisor Group, UK Health Security Agency, Birmingham, United Kingdom; 6 National Institute for Health and Care Research Health Protection Research Unit in Emergency Preparedness and Response, University of Birmingham, Birmingham, United Kingdom

**Keywords:** health services, mental health, rainfall, sunshine, weather

## Abstract

**Background:**

Weather conditions have been linked to adverse mental health outcomes, and rising concern about climate change has increased interest in these associations. However, most existing research focuses on extreme weather events, such as heatwaves, or on acute clinical outcomes, such as suicide. Evidence is more limited regarding population-level variations in mental health–related healthcare utilisation across the full range of daily weather conditions.

**Objective:**

To examine associations between daily weather conditions and unscheduled mental health–related healthcare contacts in England using large-scale national surveillance data.

**Methods:**

We conducted a retrospective observational study across nine English regions from 1 January 2014 to 31 December 2022. Outcomes were daily counts of unscheduled mental health–related contacts to emergency departments (EDs), general practice out-of-hours (GP OOH) services, and the NHS 111 telephone advice line. Weather exposures included mean daily temperature (°C), hours of full sunshine, and total daily rainfall (mm). Associations were estimated using distributed lag non-linear models at regional level and combined through two-stage multivariate meta-analysis. Models were adjusted for seasonality, long-term trends, day of week, public holidays, and population size.

**Results:**

Mental health–related unscheduled healthcare contacts showed modest but consistent associations with temperature and sunshine. Across services, relative risks (demand) increased with rising temperatures up to around 18 °C and were higher on days with fewer hours of sunshine. Sunshine demonstrated the clearest pattern, with increased utilisation on low-sunshine days across all healthcare settings. Rainfall was not consistently associated with healthcare contacts. Age-stratified analyses showed a U-shaped relationship between temperature and ED attendances among adults aged over 64 years, with higher utilisation during both colder and warmer conditions. Overall variations in daily healthcare demand were modest, typically within ±10–20% of baseline levels.

**Conclusion:**

In England, short-term variations in temperature and sunshine are associated with changes in unscheduled mental health–related healthcare utilisation, whereas rainfall shows little consistent effect. Although effect sizes were modest, these findings highlight the role of everyday weather conditions in influencing mental health–related healthcare demand and may support planning and preparedness efforts for mental health services under current and future climate conditions.

## Introduction

Mental health conditions are a leading contributor to global disease burden and affect approximately one billion people worldwide (1). In addition to substantial impacts on quality of life, mental health problems place considerable demands on healthcare systems and are projected to generate increasing economic costs over coming decades. Understanding factors that influence fluctuations in mental health–related healthcare demand is therefore an important public health priority.

Environmental and meteorological conditions have long been hypothesised to influence mental health and wellbeing. A growing body of literature has examined associations between weather variable, most commonly temperature, and a range of mental health outcomes. These effects appear to be more pronounced among certain population groups, such as older adults, women, and adolescents.

However, several important gaps remain in the evidence base. First, much of the existing research focuses on extreme weather events, whereas less is known about the impact of day-to-day variations in typical weather conditions on mental health–related outcomes. A recent UK Health Security Agency (UKHSA) report shows that even routine, non-extreme weather, particularly warmer-than-usual days and nights, can increase psychological distress, mental health service use, symptom severity in vulnerable groups, and experiences of climate-related worry, with sleep disruption acting as a key mechanism (2). An earlier UKHSA report suggests that climate change is shifting the baseline of “everyday weather”, increasing exposure to warm days and muggy conditions, all of which may affect health before extreme thresholds are even reached (3).

Second, many studies examine specific clinical diagnoses or acute outcomes, often using hospital admissions or mortality data, which capture only a subset of the broader population experiencing mental distress. Third, relatively few studies have examined multiple weather exposures simultaneously, despite the likelihood that temperature, sunlight, and precipitation may exert overlapping or interacting influences (4, 5).

Evidence relating to sunshine exposure and mental health is limited. While reduced sunlight has been linked to depressive symptoms and seasonal affective disorder, particularly through disruption of circadian rhythms, most studies rely on self-reported outcomes or focus on acute endpoints such as suicide (6–8). Similarly, research on rainfall has largely concentrated on extreme events such as flooding, with inconsistent findings regarding associations between routine precipitation and mental health (9).

An additional limitation of the literature is the lack of population-level analyses of mental health–related healthcare utilisation. Healthcare contacts reflect not only underlying symptom burden but also help-seeking behaviour, access to services, and perceived need for support. Examining patterns of healthcare utilisation across different points of access—such as telephone advice services, general practice out-of-hours (GP OOH) services, and emergency departments (EDs) may therefore provide valuable insight into how environmental conditions influence demand for mental health support at the population level (10, 11).

Here, we examined associations between daily weather conditions and unscheduled mental health–related healthcare utilisation across the National Health Service (NHS) in England using large-scale national syndromic surveillance data. Specifically, we investigated the short-term effects of ambient temperature, hours of full sunshine, and rainfall on daily counts of unscheduled mental health–related contacts to NHS 111, GP OOH services, and EDs over a nine-year period. By applying distributed lag non-linear models and multivariate meta-analysis across nine English regions, we sought to characterise non-linear, delayed, and regionally heterogeneous associations across the full range of typical weather conditions.

## Methods

### Data sources and study population

Daily anonymised and aggregated healthcare contact data were extracted from three national syndromic surveillance systems routinely operated by the UKHSA (12). Data covered the period from 1 January 2014 to 31 December 2022 and included contacts to the NHS 111 telephone advice service, GP OOH services, and EDs (**Table 1**; **Supplementary Figure 1**).

**Table 1:** Summary of NHS 111 calls, GP OOH contacts and ED attendances for mental health conditions in England 2014-2022 (All values are 1x10^3^).

Variable	Detail	NHS 111 (Total:1620)	(%)	ED (Total 1,980)	(%)	GP OOH (Total 1,023)	(%)
Region	East Midlands	177	(10.9)	187	(9.5)	90	(8.8)
	East of England	119	(7.4)	213	(10.8)	124	(12.1)
	London	210	(12.9)	283	(14.3)	200	(19.6)
	North East	92	(5.7)	66	(3.3)	9	(0.9)
	North West	182	(11.3)	311	(15.7)	230	(22.5)
	South East	265	(16.3)	277	(14.0)	165	(16.1)
	South West	202	(12.4)	191	(9.6)	69	(6.8)
	West Midlands	189	(11.6)	211	(10.6)	101	(9.9)
	Yorkshire and Humber	185	(11.4)	240	(12.1)	36	(3.5)
Age Group	Age group 0-14	34	(2.1)	102	(5.2)	117	(11.5)
	Age group 15-44	933	(57.6)	1144	(57.8)	490	(47.9)
	Age group 45-64	457	(28.2)	442	(22.3)	223	(21.8)
	Age group > 64	196	(12.1)	292	(14.8)	193	(18.9)
Sex	Male	765	(47.2)	941	(47.7)	418	(40.8)
	Female	855	(52.8)	1030	(52.3)	605	(59.1)
Diagnosis group	Alcohol	60	(3.7)	443	(22.4)	33	(3.2)
	Anxiety	*		309	(15.6)	246	(24.1)
	Depression	*		335	(16.9)	83	(8.1)
	Mental Health problems	1416	(87.4)	*		*	
	Other	*		415	(21.0)	588	(57.5)
	Overdose	*		478	(24.2)	*	
	Self-harm	54	(3.4)	*		46	(4.4)
	Sleep difficulties	90	(5.5)	*		27	(2.6)

*Not specifically coded.

Unscheduled mental health–related contacts were defined as contacts in which a clinical diagnosis, triaged call Pathway, or symptom code recorded at the point of contact related to a mental health presentation (11, 12). NHS 111 contacts included calls triaged to NHS Pathways for mental health problems, deliberate self-harm, alcohol intoxication, or sleep difficulties, using the first Pathway selected by the call handler (13). GP OOH contacts were identified using Read codes indicating anxiety, depression, self-harm, or sleep difficulties. ED attendances were identified using SNOMED-CT diagnosis codes for mental health conditions specifically anxiety, depression, self-harm and alcohol intoxication. For periods prior to April 2018, when coding systems differed, the triage code indicative of similar mental health presentations was used.

Healthcare contacts were aggregated by day and region. Region was assigned using patient postcode district for NHS 111 and healthcare service provider postcode for GP OOH and ED data. Age group and sex were extracted to allow stratified analyses.

### Weather data

Daily minimum and maximum air temperature and total daily rainfall were obtained from the HadUK-Grid dataset, mapped to one of nine governmental regions and averaged to derive regional mean temperature and rainfall (14, 15). Sunshine data were obtained from the Met Office Integrated Data Archive System (MIDAS), using automated weather stations with complete records over the study period (16). For each region, the station closest to the geographical centre was selected.

### Statistical analysis

Associations between weather exposures and healthcare utilisation were estimated using a two-stage modelling approach following Gasparrini et al. (17). In the first stage, region-specific generalised linear models with quasi-Poisson distributions were fitted to daily counts of mental health–related healthcare contacts. Distributed lag non-linear models were used to capture non-linear and delayed associations, with weather exposures modelled as moving averages over lag days 0–6. We used this lag period to focus on the short-term effects of ‘usual’ weather patterns across a range of mental health conditions. Evidence suggests that these effects differ between non-extreme and extreme weather exposures with the former exerting short-lag effects on mental health outcomes, typically occurring on the same day or within a few days and accumulating over short periods (e.g. 0–7 days) (18–20). This is in contrast to extreme weather events which have immediate impacts followed by psychological morbidity over weeks to months (21, 22).

Models were adjusted for long-term trends and seasonality using smooth functions of time. Day of week and public holidays were controlled for, and regional population size was included as a log offset. A binary indicator distinguishing pre- and post-April 2018 ED data collection periods was included. Multicollinearity between weather predictors was assessed using variance inflation factors (23, 24).

In the second stage, region-specific coefficients were generated using multivariate meta-analysis to derive pooled exposure–response relationships and to quantify between-region heterogeneity. Cochran’s Q-test was used to evaluate inter regional heterogeneity and the *I^2^* statistic to quantify the proportion of total variation across studies due to heterogeneity rather than chance.

Analyses were conducted separately for each healthcare system, with additional stratification by age group, sex, and diagnostic category. For each system multiple models were produced. The main models pooled all mental health-related data into an ‘all-MH’ indicator. A second set of models were then produced subdividing mental health-related data into specific diagnosis codes (**Supplementary Table 1**).

Sensitivity analyses were performed by excluding data from the COVID-19 pandemic years (2020–2021) and comparing model fit using Akaike Information Criterion values.

## Results

Across the study period, over 4.6 million mental health–related healthcare contacts were recorded across the three surveillance systems. NHS 111 accounted for the largest volume of contacts, followed by ED attendances and GP OOH contacts. Most contacts occurred among individuals aged 15–44 years, with slightly higher utilisation among females (**Table 1**).

### Temperature

**Figure 1:**
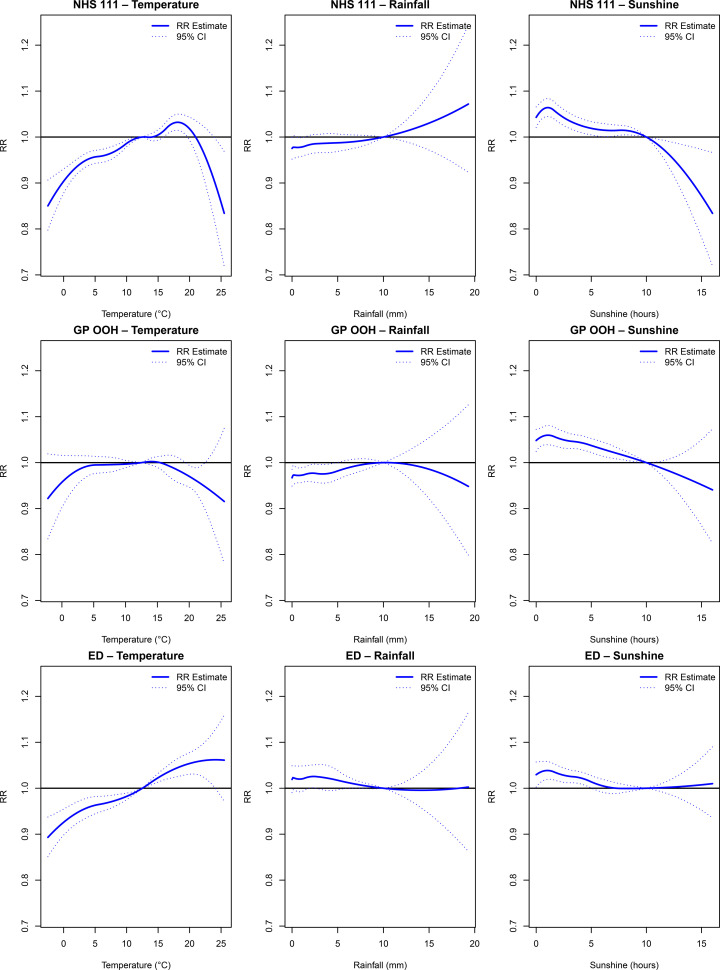
Multivariable model pooled exposure-response relationship in relative risk between mean temperature (left), rainfall (centre column) and hours of full sunshine (right) and calls to NHS 111 (top), GP out of hours contacts (middle) and emergency department attendances (bottom) in nine English regions, 2014-2022. The dashed lines represent 95% confidence intervals. Reference at 12.5 °C, 10mm and 10 hours respectively.

Multivariable results for mean temperature are presented in **Figure 1** (left column). Pooled exposure–response relationships indicated modest, non-linear associations between mean daily temperature and mental health–related healthcare utilisation. For NHS 111 calls and ED attendances, relative risks were lower at colder temperatures and increased gradually as temperature rose up to approximately 18 °C, before flattening or declining at higher temperatures. Associations for GP OOH contacts were weaker and largely flat.

Relative to the reference temperature of 12.5 °C (defined by the data presented to the model), minimum relative risks were observed at approximately −3 °C for NHS 111 calls (RR 0.83, 95% CI 0.78–0.90) and −2.5 °C for ED attendances (RR 0.90, 95% CI 0.85–0.94). Maximum effects were observed at 18.1 °C for NHS 111 calls (RR 1.03, 95% CI 1.01–1.05) and 24.7 °C for ED attendances (RR 1.07, 95% CI 0.99–1.16) (**Figure 1**). The relative difference between minimum and maximum relative risks was approximately 20% for NHS 111 and 17% for ED services.

Age-stratified analyses showed little deviation from pooled estimates (**Supplementary Figure 2**) for NHS 111. For ED attendances, individuals aged over 64 years demonstrated a U-shaped association, with higher utilisation during both colder and warmer conditions, (**Supplementary Figure 2**).

**Figure 2:**
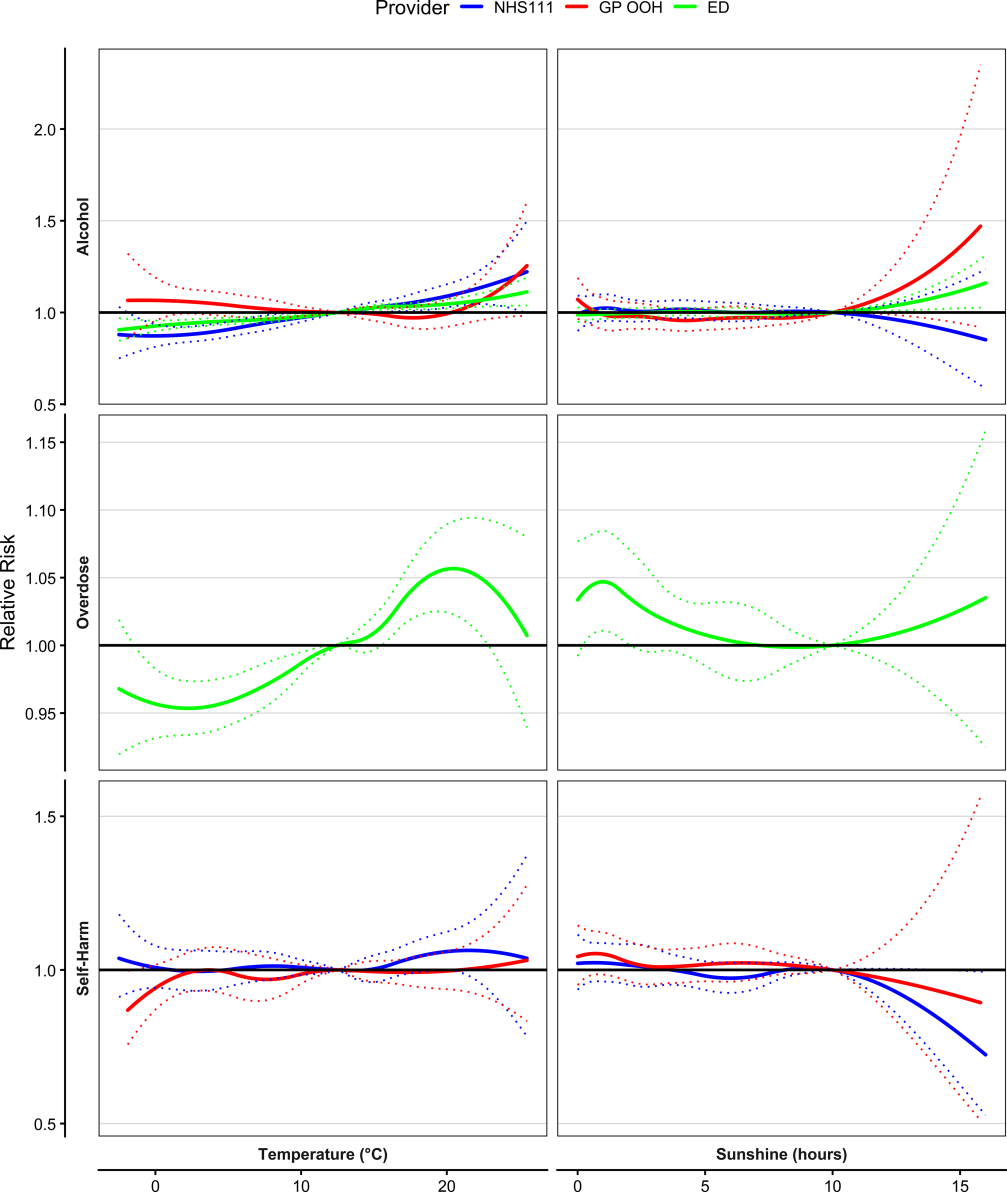
Multivariable model pooled exposure-response relationships between mean temperature (left) and hours of full sunshine (right) and mental health conditions related to alcohol, overdose and self-harm. Solid lines represent pooled estimates. Dashed lines represent 95% confidence intervals. Please note that y axes vary.

**Figure 3:**
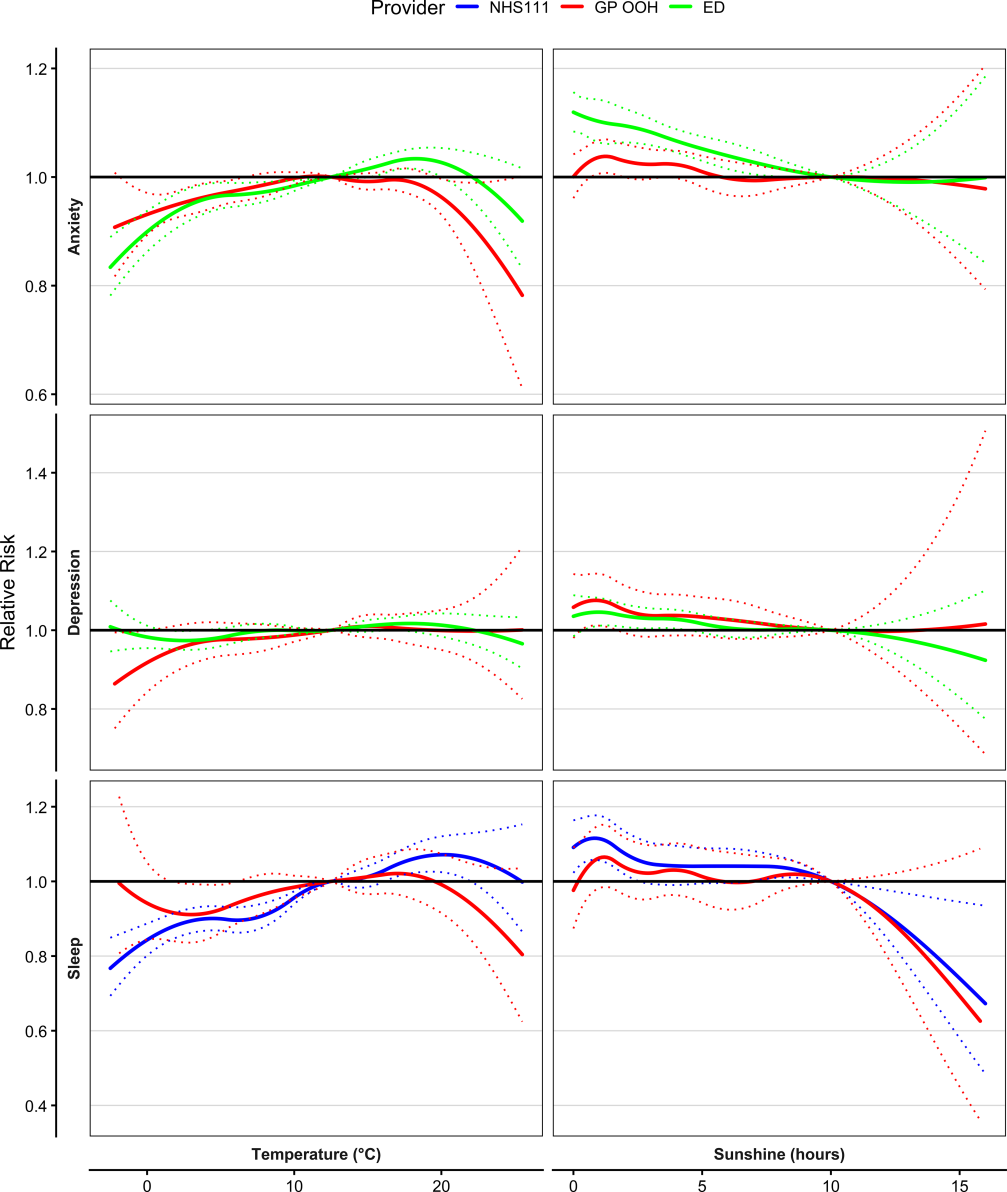
Multivariable model pooled exposure-response relationships between mean temperature (left) and hours of full sunshine (right) and mental health conditions related to anxiety, depression and sleep. Solid lines represent pooled estimates. Dashed lines represent 95% confidence intervals. Please note that y axes vary.

The all-MH data is a mixture of different conditions, hence an analysis of specific codes of mental health conditions was undertaken. This showed that higher temperatures were associated with increased NHS 111 calls and ED attendances for alcohol-related conditions and NHS 111 calls about overdoses increased with temperature (**Figure 2**). **Figure 2** also indicates no clear associations between temperature and self-harm. Lower temperatures were associated with reduced GP OOH contacts and ED attendances for anxiety-related conditions, but no clear associations were observed between temperature and contacts for self-harm or depression (**Figure 3**). NHS 111 contacts for sleep disorders increased at higher temperatures (**Figure 3**).

### Rainfall

Multivariable results for rainfall are shown in **Figure 1** (middle column). Rainfall showed no consistent association with mental health–related healthcare utilisation across services. Most pooled estimates were close to null, with confidence intervals spanning one. Stratified analyses by age and sex showed little deviation from pooled effects (**Supplementary Figure 2** and **3**), and no consistent associations were observed for specific diagnostic categories (data not shown).

### Sunshine

Multivariable results for sunshine displayed the most consistent associations across all healthcare systems and are presented in **Figure 1** (right column). Fewer hours of sunshine were associated with higher relative risks of mental health–related healthcare contacts for NHS 111, GP OOH, and ED services. Relative to a reference of 10 hours of sunshine per day (defined by the data presented to the model), the greatest increases in utilisation were observed on days with minimal sunshine (NHS 111: RR 1.06, 95% CI 1.04–1.08; GP OOH: RR 1.06, 95% CI 1.04–1.08; ED: RR 1.04, 95% CI 1.02–1.07).

Age-stratified analyses indicated broadly similar patterns across age groups, although increases in ED utilisation were most pronounced among individuals aged 45–64 years. (**Supplementary Figure 2**). Stratification by sex showed little difference from pooled estimates (**Supplementary Figure 3**).

Analyses of specific mental health conditions (**Figure 2**, **3**) showed no clear associations with sunshine hours overall. However, fewer sunshine hours were associated with increased GP OOH and ED contacts for anxiety and depression (**Figure 3**).

### Regional heterogeneity

Heterogeneity across second-stage multivariate models was generally low to moderate. Sunshine demonstrated the most consistent effects across all syndromic systems (I² ≤35%), with no evidence of heterogeneity in NHS 111 data (p=0.17). Temperature showed modest heterogeneity, particularly within GP OOH and ED data (I² ≈33%; p ≤ 0.02), while rainfall exhibited the greatest variability, most notably in emergency department data where substantial heterogeneity was observed (I²=56.3%; p<0.001). Across all predictors, heterogeneity was consistently higher for emergency department data compared with NHS 111 and GP out-of-hours services.

### Sensitivity analysis

Excluding data from the COVID-19 pandemic years resulted in minor changes to the shape of exposure–response curves, but the overall direction and magnitude of associations remained consistent. Models including the full study period generally demonstrated lower AIC values, indicating better relative model fit (**Supplementary Table 3** and **Supplementary Figure 4**).

## Discussion

Using nine years of national UKHSA syndromic surveillance data, we found that unscheduled mental health-related healthcare utilisation in England varied modestly but significantly with short-term changes in temperature and sunshine, while rainfall showed little consistent association.

Higher relative risks of unscheduled healthcare contact were observed at increasing temperatures up to moderate levels and on days with fewer hours of sunshine i.e. they increased during periods of hot weather and low sunshine. These associations were evident across the full range of typical weather conditions and persisted after accounting for healthcare system disruptions during the COVID-19 pandemic.

Our findings are consistent with previous evidence linking ambient temperature to mental health–related outcomes, particularly studies reporting increased healthcare utilisation or symptom severity during periods of heat or cold (4, 25). UKHSA reports have identified the need for an improved understanding of the mental health impacts of climate change (3) and that climate-related mental health impacts will increase pressure on health and social care systems (2). By focusing on routine weather variability rather than extremes, this study extends existing literature and demonstrates that smaller, day-to-day fluctuations in weather are also associated with changes in mental health–related healthcare demand.

The inverse association between sunshine hours and healthcare utilisation (i.e. higher during periods of little sunshine) was the most consistent finding across services. Although fewer studies have examined sunshine directly, these results align with evidence linking reduced sunlight exposure to depressive symptoms and seasonal affective disorder (6, 8). Importantly, the outcomes examined here reflect healthcare-seeking behaviour rather than diagnosed psychiatric disorders, suggesting that reduced sunshine may be associated with changes in distress, symptom perception, or coping that increase demand for support. Rainfall showed little consistent association, supporting previous mixed findings in the literature (5, 9).

The mechanisms underlying these associations are likely to be complex and multifactorial, reflecting a combination of biological responses, psychological processes, and behavioural factors, including health-seeking behaviour and social activity patterns (26, 27). We also used an aggregate outcome variable made up of multiple mental health conditions which may have enhanced or masked effects for specific conditions. This is reflected in the all-MH categories but also in the more specific indicators such as “depression” which is still a syndrome of multiple health conditions. We note that weather may affect specific mental health conditions, such as schizophrenia, in different ways (28) In this study we focussed upon short-term impacts of weather upon mental health outcomes, but this approach may not capture cumulative exposure response events or longer-term mental health changes. As this study examined healthcare contacts rather than clinical diagnoses, findings do not provide evidence of changes in the incidence or prevalence of mental disorders.

A key strength of this study is its scale. We analysed nine years of daily syndromic surveillance data routinely captured and monitored by UKHSA, encompassing over 4.6 million mental health–related healthcare presentations, making this one of the largest studies of its kind to date. The analysis integrated multiple points of access to unscheduled care, including a national telephone advice service (NHS 111), GP OOH, and ED attendances. Our multivariable modelling framework simultaneously examined the effects of daily mean temperature, hours of full sunshine, and daily rainfall, while controlling for seasonality, long-term temporal trends, and day-of-week effects. In addition, the modelling approach explicitly accounted for non-linear exposure–response relationships and delayed effects, which are known to be context dependent and vary geographically, and which have been identified as key methodological recommendations in recent systematic reviews (4, 5).

A further important consideration is that the mental health data used in this study represent requests for healthcare or advice rather than confirmed clinical diagnoses. This is a key strength of the study, as formal diagnoses are likely to constitute only a subset of total mental health–related healthcare contacts. The data therefore capture a broad spectrum of help-seeking behaviour, including presentations that may not reach the threshold for a formal diagnosis but nonetheless generate demand for mental health services and associated resources. Although healthcare-seeking behaviour can be influenced by external factors such as media reporting (29), it is unlikely that such influences would be systematically associated with daily weather conditions and therefore unlikely to bias the associations reported here. The findings reflect environmental conditions as experienced in England, characterised by a mean daily ambient temperature of 12.5 °C, daily rainfall of 10 mm, and daily sunshine duration of 10 hours over the study period. Accordingly, the results pertain to healthcare-seeking behaviour within England in the context of a nationalised healthcare system that is free at the point of use.

England is a relatively small country, and we subdivided our analysis into nine smaller regions. Whilst broad differences in weather likely exist across and within English regions, heterogeneity across second-stage multivariate models was generally low to moderate suggesting that the estimated associations are broadly consistent across regions. However, heterogeneity was consistently higher for emergency department data, which may reflect regional arrangements for emergency care provision, greater variability in more severe presentations, differences in healthcare-seeking behaviour, or increased sensitivity to unmeasured contextual factors.

Although effect sizes were modest (2-19% depending on the indicator and predictor variable), even small relative changes in healthcare utilisation may translate into meaningful fluctuations in service demand at a national scale. The indicators used are for ‘first contact’ with health services and do not reflect consequential pressure on health and social services associated with the complexity and possibly chronic nature of mental health conditions requiring secondary or ongoing scheduled treatment. In addition, these trends may not be reflective of healthcare seeking outside of the NHS and of individuals who choose not to seek any help.

We note the potential for psychological and logistical effects associated with the COVID 19 pandemic but this was not the focus of this study and would only bias our results if these psychological and logistical effects were associated with weather which we consider unlikely. However, the data shown in **Figure 1** of the **Supplementary Material** show short term interruptions to surveillance systems during the pandemic and a return to broadly expected levels afterwards. The sensitivity analysis also shows that the results were largely unaffected by the pandemic period, likely due to its short duration relative to the entire study period. Other research has used similar data to explore the impact of the COVID 19 pandemic upon mental health presentations (10).

These findings may inform short-term service planning in NHS healthcare services and contribute to preparedness efforts that account for weather-related variation in mental health–related demand. Over longer time scales similar approaches may help improve understanding of how climate change could influence patterns of healthcare utilisation, while recognising the potential for adaptation and changing vulnerability (30, 31).

Climate change is already influencing weather patterns and is expected to continue doing so over coming decades (2). In this context, the observed positive associations between ambient temperature and mental health–related healthcare utilisation warrant attention. However, rising average temperatures may not necessarily translate directly into increased mental health burden, as populations may adapt over time. At the same time, climate change is projected to increase temperature variability, which may pose greater challenges for societal adaptation. Our findings also indicate that greater sunshine duration was associated with reduced mental health–related healthcare utilisation. Projections suggest decreasing cloud cover over most continents, with increasing cloud cover in tropical regions; in temperate regions such as the UK, changes in sunshine may therefore partially counterbalance some temperature-related effects (31).

This research has several public health implications. First, improved understanding of associations between weather conditions and mental health–related healthcare demand may inform the calibration of early warning systems, particularly to support healthcare providers in emergency preparedness and resource allocation. Second, applying similar analytical approaches over longer time scales could contribute to understanding the potential impacts of climate change on mental health–related healthcare utilisation. Finally, this study highlights the value of syndromic surveillance data as a resource for monitoring mental health–related healthcare demand. The consistency of our findings with other studies using comparable methods suggests that, where such systems are available, standardised syndromic surveillance data could be applied in similar ways in other settings beyond England.

## Conclusion

This study provides evidence that short-term variations in temperature and sunshine are associated with changes in unscheduled mental health–related healthcare utilisation within NHS services in England. Higher temperatures and fewer hours of sunshine were linked to modest increases in healthcare contacts, while rainfall showed little consistent association. Although the observed effects were small in magnitude, the findings demonstrate the utility of near real-time syndromic surveillance data for monitoring fluctuations in mental health–related healthcare demand. Integrating environmental data with routine healthcare surveillance may support service planning and preparedness and offers a framework for examining how weather and longer-term climatic changes could influence patterns of mental health–related healthcare utilisation.

## Data Availability

The data analyzed in this study is subject to the following licenses/restrictions: The anonymised data used is held by UKHSA. Requests to access these datasets should be directed to https://www.gov.uk/government/publications/accessing-ukhsa-protected-data.
