# Supplementary material for: The effect of weather on unscheduled healthcare utilisation for mental health conditions in England, 2014–2022
Source: Front Psychiatry. 2026 Jun 30;17:1835204. doi: 10.3389/fpsyt.2026.1835204 (PMC13363332; doi:10.3389/fpsyt.2026.1835204)
Supplement: Supplementary file 1 [file SupplementaryFile1.docx]

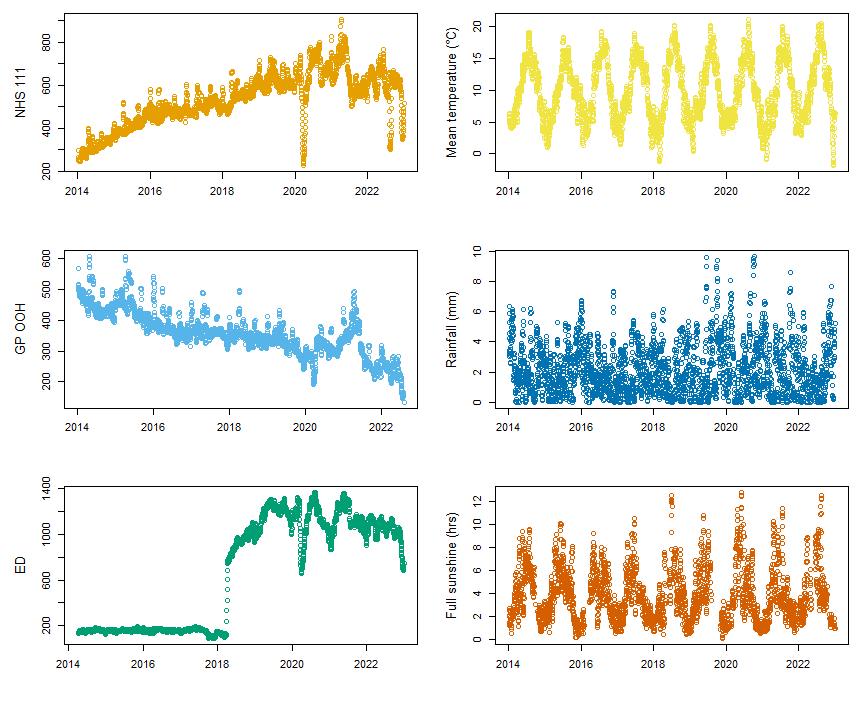
**Supplementary Figure 1. Outcome and predictor variables measured daily over time during monitoring period.**

**Notes**: Data are presented as a 7-day moving average. Date range for NHS 111 and GP OOH is January 2014-December 2022. Date range for ED is April 2014- December 2022. Left column (Top: NHS 111 calls, Centre: GP OOH contacts, Bottom: ED attendances). Right column (Top: Mean temperature, Centre: Rainfall, Bottom: Full sunshine)

**Supplementary Figure 2. Multivariable model pooled exposure-response relationships stratified by age group.**


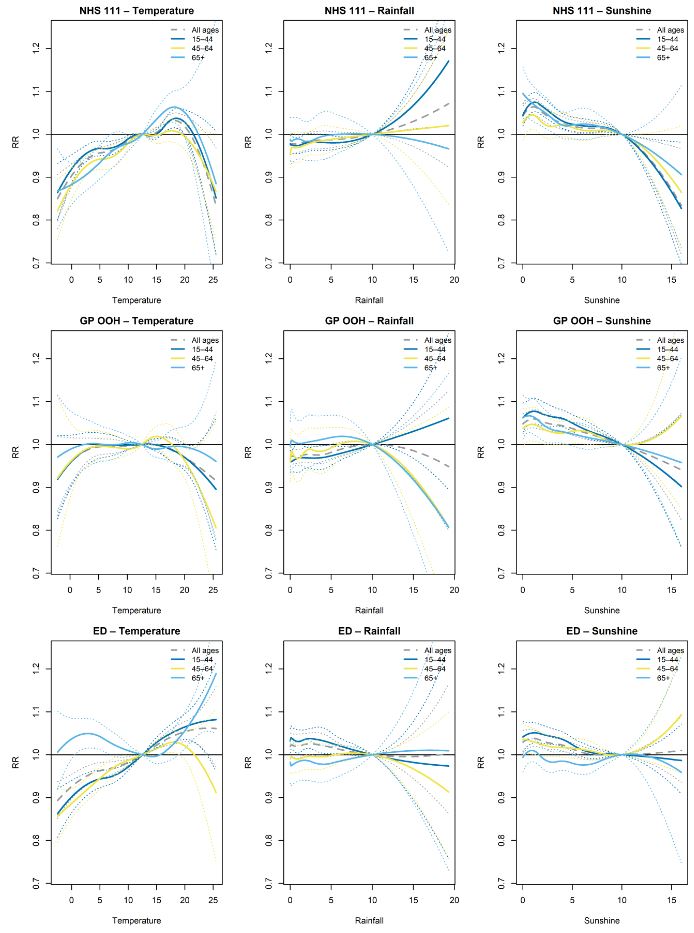


**Notes:**  Solid lines represent age group specific pooled estimates. Dotted lines represent 95% confidence intervals.

**Supplementary Figure 3. Multivariable model pooled exposure-response relationships stratified by sex.**

**
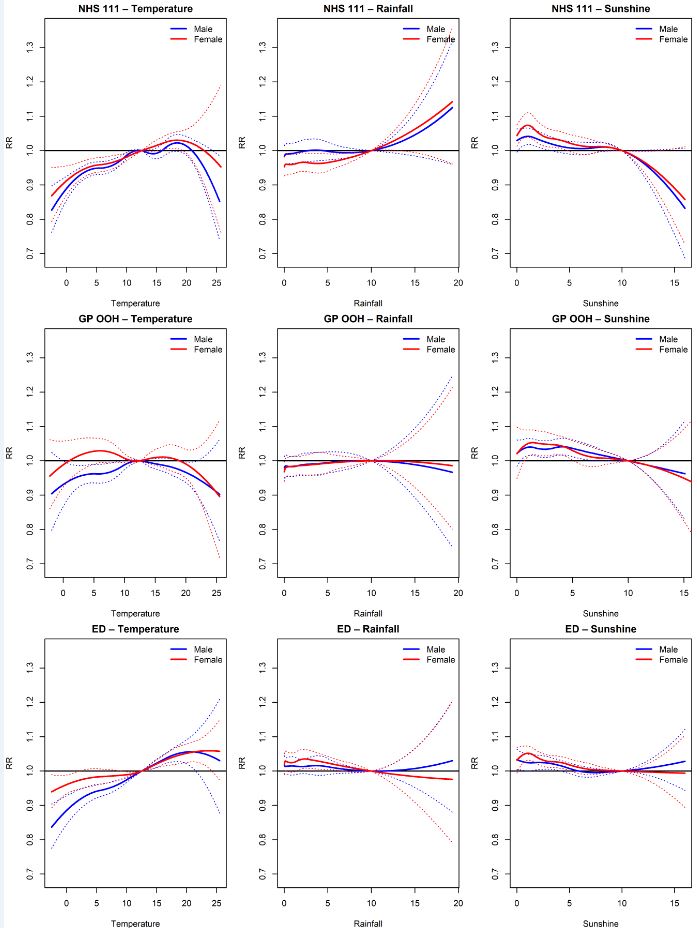
**

**Notes:**

Solid lines represent sex specific pooled estimates. Dotted lines represent 95% confidence intervals. Outcome variables: Top row: calls to NHS 111. Middle row: GP out of hours contacts. Bottom row: emergency department attendances. stratified by age group in England, 2014-2022. Predictor variables: Left: Temperature. Centre: Rainfall. Right: Sunshine. Reference at 12.5 °C, 10mm and 10 hours respectively.

**Supplementary Figure 4. Multivariable pooled exposure response relationship with and without period of COVID-19 pandemic (2020-2021).**

**
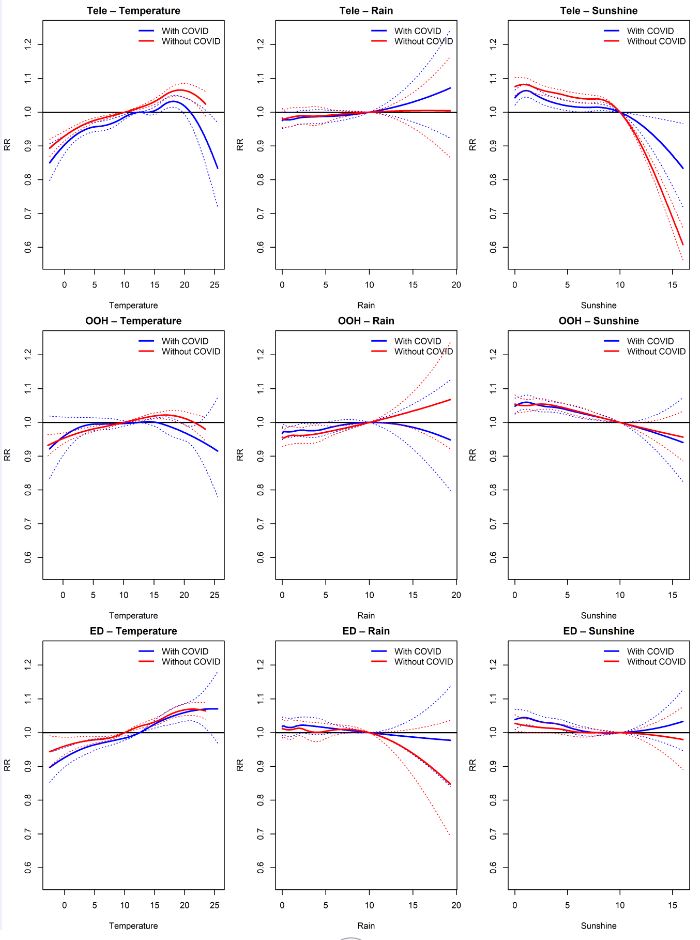
**

**Supplementary Table 1. Construction of diagnosis groups.**

| **Diagnosis group** | **Variables included** | | |
| --- | --- | --- | --- |
|  | **NHS 111*** | **GP OOH*** | **ED*** |
| **Alcohol** | Alcohol intoxication | Alcohol intoxication | Alcohol intoxication |
| **Sleep** | Sleep difficulties | Sleep difficulties/insomnia | Not recorded |
| **Self-harm** | Deliberate self-harm | Intentional poisoning  Self-harm | Not recorded |
| **Overdose** | Not recorded | Overdose | OD/poisoning/toxic |
| **Anxiety** | Not recorded | Anxiety | Anxiety |
| **Depression** | Not recorded | Depression | Depression |
| **Other** | Mental health problems | Mental health/total neuroses | All mental health |

Notes:

* See Table 1 for detail.

**Supplementary Table 2. Cochran Q test and I^2^ for second stage multivariate models based on an absolute scale.**

| **Predictor variable** | **Source** | **Cochran Q test** | | | **I-square** |
| --- | --- | --- | --- | --- | --- |
|  |  | **Q** | **df** | **p** |  |
| **Temperature** | NHS 111 | 63.2 | 48 | 0.07 | 24.0 |
|  | GP OOH | 71.4 | 48 | 0.02 | 32.8 |
|  | ED | 71.9 | 48 | 0.01 | 33.3 |
| **Rainfall** | NHS 111 | 75.4 | 48 | 0.01 | 36.3 |
|  | GP OOH | 63.8 | 48 | 0.06 | 24.7 |
|  | ED | 109.8 | 48 | 0.00 | 56.3 |
| **Sunshine** | NHS 111 | 57.4 | 48 | 0.17 | 16.4 |
|  | GP OOH | 62.9 | 48 | 0.07 | 23.7 |
|  | ED | 73.9 | 48 | 0.01 | 35.1 |

**Supplementary Table 3. Comparison of models with 2020-2021 data included (base) and excluded (1) using Akaike Information Criterion (AIC).**

| **System** | **Variable** | **AIC base** | **AIC 1** | **Δ AIC** |
| --- | --- | --- | --- | --- |
| **NHS 111** | Mean temperature | -104.8 | -102.1 | 2.7 |
|  | Rainfall | -123.8 | -104.3 | 19.4 |
|  | Sunshine | -131.3 | -92.2 | 39.1 |
| **GP OOH** | Mean temperature | -76.9 | -82.9 | -6.0 |
|  | Rainfall | -104.0 | -92.6 | 11.4 |
|  | Sunshine | -107.1 | -95.4 | 11.8 |
| **ED** | Mean temperature | -150.5 | -143.6 | 6.9 |
|  | Rainfall | -127.2 | -110.5 | 16.7 |
|  | Sunshine | -149.4 | -126.5 | 22.9 |
